# Lectures on Medical Etiquette

**Published:** 1909-04-17

**Authors:** 


### Lectures on Medical Etiquette.

It is almost a truism that, Medical Ethics and Medical Etiquette, those powerful factors in the professional and social life of every medical man, stand in increasing need of codification, if not of simplification or entire revision. The rules of conduct governing our relations one with another, and with our patients and their friends and the public generally, have many of them arisen naturally out of our peculiar position in the social machine. It may indeed be said with justice of at least a majority of these rules that their essential object is the maintenance of the dignity and honour of the profession, and the well-being of the public to which it ministers. That these two worthy objects are now and then difficult to reconcile in particular instances is an inevitable drawback, which should not however excuse the abandonment of any sensible scheme for the definition and systematisation of the Laws of Medical Etiquette. The fundamental rule of civilised human conduct "Do unto others as you would they should do unto you"—however much obscured by needless forms and usages—still lies at the bottom of most of our professional observances. Even those rules which to the lay mind seem the most exasperating and capricious are yet capable of honest defence. Scarcely less common than the traditional layman's sneers at the expense of medical etiquette—which too often seems to him a senseless incubus of red-tape and ritual, designed to come between patient and doctor—are the legitimate professional complaints at the uncertainty and lack of authoritative ruling with which the ethics and etiquette of our profession are encumbered. Situations daily arise in actual practice or in the business aspects of medicine, which are complicated by considerations of medical etiquette or occur solely through ignorance or misinterpretation of its unwritten laws. Newly qualified men enter practice entirely uninstructed in the principles of medical ethics; and, even when brought up in a professional household, their ideas upon medical etiquette are for the most part crude, hazy and confused. The better class of medical student (happily more and more common nowadays) when he qualifies and begins his life work, needs no lectures or demonstrations upon the principles and practice of "playing the game." But there is more than that in medical ethics, just as there is more in medical etiquette than a nice familiarity with the procedure of after-dinner-party calls, and the correct management of the hat and umbrella. With this in mind, an anonymous "G. P." wrote recently to the *St. Bartholomew's Hospital Journal* suggesting that one or two lectures should be given in the medical school of his old hospital at the end of each session, on Medical Ethics and Etiquette, to those students who have finished their course and are just about to qualify. He proposes, very pertinently as it seems to us, that these lectures should be given by "some well-known general practitioner of high standing and long experience; and not by a consultant on the hospital staff, whose career has not enabled him to gain experience of the petty annoyances and temptations to diverge from the straight path, which are so often cropping up in the life of the general practitioner." He maintains finally that "the opinion is almost universal among general practitioners that instruction to young medical men in these subjects is desirable"; and, with the reservation that this idea has hitherto to a large extent been felt subconsciously rather than expressed openly, we agree. In the following issue (that for April) we observe a note to the effect that a recommendation has been sent up from the council of the Students' Union of St. Bartholomew's Hospital, asking the Medical School Committee to arrange for lectures to be delivered by prominent general practitioners upon the lines suggested. The proposal has our warm sympathy and support; and we hope that the experiment will be permitted by the authorities and that such success will attend the early lectures that other medical schools will follow suit.

